# Enteric Methane Emission and Milk Component Profiles of Holstein and Jersey Cows: A Respiration Chamber Study

**DOI:** 10.3390/ani16182898

**Published:** 2026-09-15

**Authors:** Jeongkuk Park, Jun Sik Eom, Dong Hyeon Kim, Seong Min Park, Su Bin Han, Younglae Kim, Chang-Gwon Dang, Dong-Hyun Lim

**Affiliations:** 1 Dairy Science Division, National Institute of Animal Science, Rural Development Administration, Cheonan 31000, Republic of Korea; pjk1028@korea.kr (J.P.); kimdh3465@korea.kr (D.H.K.); bek9love@korea.kr (S.M.P.); sbhan1229@korea.kr (S.B.H.); yl1342@korea.kr (Y.K.); gkgkgki@korea.kr (C.-G.D.); 2 Precision Animal Nutrition Division, National Institute of Animal Science, Rural Development Administration, Wanju 55365, Republic of Korea; eomjs0606@korea.kr

**Keywords:** Holstein, Jersey, enteric methane, respiration chamber, milk components, methane intensity

## Abstract

Holstein and Jersey dairy cows differ in body size, feed intake, milk production, and milk composition, which may influence the interpretation of enteric methane (CH_4_) emissions. This study compared production traits, milk composition, and respiration chamber-measured CH_4_ emissions between 10 Holstein and 10 Jersey cows. Repeated measurements were averaged within cow, and breed differences were evaluated using independent two-sample *t*-tests. Sensitivity analyses additionally adjusted for age and days in milk. Holstein cows had greater body weight, dry matter intake, milk yield, fat- and protein-corrected milk, and daily CH_4_ production than Jersey cows. Jersey cows had higher milk fat and protein concentrations, whereas daily milk fat yield did not differ significantly between breeds. No significant breed differences were detected in CH_4_ production per unit of dry matter intake, milk yield, or fat- and protein-corrected milk. In the unadjusted analysis, Jersey cows had lower CH_4_ production per unit of milk fat yield, but the breed difference was inconclusive after adjustment for age and days in milk. Methane production per unit of milk protein or lactose yield did not differ significantly between breeds. These findings indicate that breed comparisons of enteric CH_4_ depend on the basis used to express emissions, while the component-specific ratios should be interpreted as exploratory animal-level measures.

## Simple Summary

Holstein and Jersey cows differ in body size, feed intake, milk production, and milk composition, and these differences may affect how methane emissions are compared between the breeds. This study measured methane produced by 10 Holstein and 10 Jersey cows using respiration chambers and compared emissions in relation to feed intake, milk production, and the amounts of fat, protein, and lactose produced in milk. Holstein cows consumed more feed, produced more milk, and emitted more methane per day than Jersey cows. Jersey cows produced milk with higher fat and protein concentrations. However, methane emissions relative to feed intake or corrected milk production did not differ significantly between the breeds. In the unadjusted comparison, Jersey cows produced less methane per unit of milk fat, but the breed difference was inconclusive after adjustment for age and days in milk. No breed differences were detected when methane was expressed relative to milk protein or lactose production. These findings show that comparisons of methane emissions between dairy breeds can change depending on the production measure used. The component-based comparisons are exploratory and should not be interpreted as showing an overall methane advantage for either breed.

## 1. Introduction

Enteric methane (CH_4_) is generated as a natural consequence of microbial fermentation in the rumen and represents an important source of greenhouse gas emissions from dairy cattle [1]. Daily CH_4_ production is influenced by dry matter intake (DMI), dietary composition and digestibility, body weight (BW), and milk production, and can be expressed as absolute production per cow, yield relative to DMI, or intensity relative to productive output [2]. These metrics are complementary rather than interchangeable. Daily CH_4_ production quantifies emissions per animal, CH_4_/DMI relates emissions to feed intake, and milk-based CH_4_ intensity relates emissions to productive output.

Dairy breeds differ in body size, feed intake, milk production, and milk composition. A meta-analysis of direct breed comparisons and data from commercial dairy herds showed that Holstein cows generally produce more milk and milk components per cow, whereas Jersey cows produce milk with higher fat and protein concentrations [3,4]. Higher component concentrations, however, do not necessarily result in greater daily component yields because component output is determined jointly by milk volume and component concentration. Fat- and protein-corrected milk (FPCM) and individual component yields therefore provide different production bases for evaluating CH_4_ by accounting for distinct combinations of milk volume and composition [5].

Previous direct comparisons have not produced a consistent breed ranking across CH_4_ metrics. Holstein cows have been reported to produce more CH_4_ per day than Jersey cows without corresponding breed differences in CH_4_/DMI or CH_4_/FPCM. Other comparisons have identified breed differences in CH_4_/DMI and breed-dependent responses to forage-to-concentrate ratio or high-concentrate diets [6,7,8]. These findings indicate that differences in daily CH_4_ production cannot be assumed to persist when emissions are expressed relative to feed intake or corrected milk production.

The available direct breed comparisons have primarily evaluated daily CH_4_ production, CH_4_/DMI, and milk- or corrected-milk-based CH_4_ intensity, while CH_4_ relative to combined milk solids has also been examined in genetically divergent grazing dairy cows [9]. However, simultaneous comparison of CH_4_ relative to separate milk fat, protein, and lactose yields remains limited. These component-specific ratios were included as exploratory animal-level metrics to test whether the observed breed comparison changed when daily CH_4_ production was normalized to different measured milk outputs. They were evaluated alongside established CH_4_ production, yield, and corrected-milk intensity metrics rather than as alternatives to those established measures.

The objective of the present study was to compare Holstein and Jersey cows with respect to BW, DMI, milk yield, FPCM, milk composition, daily milk component yields, and respiration chamber-measured CH_4_ production. Methane traits were evaluated as daily CH_4_ production and relative to BW, DMI, milk yield, FPCM, milk fat yield, milk protein yield, and milk lactose yield. The study aimed to characterize the distinct production and CH_4_ profiles of Holstein and Jersey cows and to determine whether the interpretation of breed differences changed according to the basis used to express CH_4_.

## 2. Materials and Methods

### 2.1. Study Design and Animals

This study used repeated respiration chamber measurements obtained from lactating Holstein and Jersey cows at the National Institute of Animal Science, Rural Development Administration, Cheonan, Republic of Korea. All cows were at least 2 years of age (Holstein, 65.62 ± 22.64 months; Jersey, 35.14 ± 6.72 months; mean ± standard deviation) and were lactating during the experimental periods (days in milk: Holstein, 194.77 ± 84.10; Jersey, 149.96 ± 90.97; mean ± standard deviation). Parity also differed between breeds: eight Holstein cows were in second parity, one was in third parity, and one was in fourth parity, whereas nine Jersey cows were primiparous and one was in second parity. Outside the respiration chamber periods, cows were housed in a conventional dairy barn at the National Institute of Animal Science. The dataset included 10 Holstein cows and 10 Jersey cows, with a total of 64 and 90 daily measurement records, respectively. Each cow could contribute up to nine daily measurement records across the three repeated measurement periods. Holstein cows contributed 3 to 9 records because measurements were discontinued within a chamber period when a cow showed poor adaptation to the respiration chamber, primarily manifested as reduced appetite and feed intake. Consequently, 26 scheduled Holstein daily measurement records were not obtained. No completed daily measurement records were excluded post hoc on the basis of production level or measurement-period assignment.

Each cow was scheduled for up to three repeated respiration chamber measurement periods, each preceded by a 2-week dietary adaptation period. The measurement periods were not fully balanced between breeds across calendar time. Cows remained in the respiration chambers for approximately 96 h, and the initial 24 h after chamber entry were excluded as an in-chamber adaptation period. Only records containing complete information on body weight, dry matter intake, milk yield, milk composition, milk component yields, fat- and protein-corrected milk, and daily CH_4_ production were included in the analysis. The Institutional Animal Care and Use Committee of the National Institute of Animal Science (NIAS 2023-0608) approved all animal procedures.

### 2.2. Animal Management and Feed Intake Measurement

During each respiration chamber measurement period, cows were offered a predetermined amount of total mixed ration (TMR) according to the nutrient requirements specified in the Korean Feeding Standard for Dairy Cattle [10]. For Holstein cows, the daily feed allowance was set at 22.0 kg of dry matter (DM) and 32.5 Mcal of net energy for lactation (NEL), based on a target milk yield of 30 kg/day with 4.0% milk fat and 3.2% milk protein. Jersey cows received the same TMR formulation at approximately 15.4 kg DM/day, corresponding to approximately 70% of the Holstein allowance based on their calculated nutrient requirements. The TMR was offered twice daily at 09:30 and 16:30, and water was provided ad libitum.

Individual feed intake was determined by weighing the amounts of feed offered and refused. The calculated feed allowances were used as reference amounts, and TMR was offered in excess to allow refusals. The mean proportion of offered feed refused was approximately 20.5% for Holstein cows and 11.5% for Jersey cows. Thus, DMI was not constrained by the calculated feed allowance. Daily TMR intake was converted to a DM basis using the analyzed DM concentration of the TMR. The chemical composition of the TMR was analyzed by the Foundation of Agricultural Technology Commercialization & Transfer (Iksan, Republic of Korea) using AOAC International procedures for DM, crude protein, ether extract, crude fiber, and crude ash and the Van Soest method for neutral detergent fiber and acid detergent fiber. The ingredient composition and analyzed chemical composition of the TMR are presented in Table 1. Body weight was measured statically before entry into the respiration chamber and after completion of the measurement period using a fixed steel platform scale equipped with a CI-200A weighing indicator (CAS, Yangju, Republic of Korea).

### 2.3. Respiration Chamber Measurement

Enteric CH_4_ production was measured using four respiration chambers developed at the National Institute of Animal Science. The configuration and validation of the respiration chamber system have been described previously by Eom et al. [12]. The chambers operated as open-circuit flow-through systems. Air from the surrounding chamber laboratory entered each chamber through air inlets, and chamber air was continuously drawn through the exhaust line by a blower. The exhaust airflow from each chamber was maintained at 700 L/min using a mass flow meter (FM153BN, ENBAC, Gumi, Republic of Korea). A subsample of the exhaust air from each chamber was drawn through 6-mm tubing, passed through a filter and a gas drying unit (W.A. Hammond Drierite Co., Ltd., Xenia, OH, USA) containing 8-mesh indicating Drierite (Drierite Absorbent, Xenia, OH, USA), and sequentially delivered to a CH_4_ analyzer (Horiba Ltd., Kyoto, Japan) at a sampling flow rate of 6 L/min.

The CH_4_ analyzer was zero-calibrated using 100% N_2_ before calibration with a standard gas containing 804.4 μmol/mol CH_4_. Chamber recovery tests were conducted by injecting a gas containing 25% CH_4_ balanced with N_2_ at 0.5 L/min using a mass flow controller (AALBORG, Orangeburg, NY, USA). Measurements were recorded at 10 s intervals for approximately 24 h before and after the experimental periods. The chamber-specific mean CH_4_ recovery rates ranged from 98.51 to 99.85%.

For routine CH_4_ measurements, air from each chamber was sampled for 150 s, followed by a 150 s purge period, resulting in a total measurement cycle of 300 s per chamber. Daily CH_4_ production was calculated considering chamber airflow, air temperature and humidity, and the measured CH_4_ concentration. The CH_4_ volume was converted from L/day to g/day using the standard molar gas volume of 22.414 L/mol, the molar mass of CH_4_ of 16.04 g/mol, and the chamber-specific recovery rate.

The chambers were opened twice daily at 09:30 and 16:30 for milking, collection of feed refusals, TMR feeding, and manure removal. Measurements affected by chamber opening were identified by visual inspection of the CH_4_ concentration trace and excluded until the concentration returned to the surrounding stable pattern. This typically resulted in the exclusion of 2–4 measurement cycles. Daily CH_4_ production was then normalized to 24 h based on the remaining valid measurement time. During the experimental periods, the mean temperature and relative humidity in the chamber laboratory were 20.81 ± 2.38 °C and 59.04 ± 17.16%, respectively, while those inside the respiration chambers were 19.56 ± 2.54 °C and 77.86 ± 10.58%, respectively.

### 2.4. Milk Sampling, Composition Analysis, and Derived Variables

Cows were milked twice daily in the respiration chambers at 09:30 and 16:30 using a portable bucket-type milking machine (Model DVI-AS, Melasty Milking Machines, Bursa, Türkiye). Individual milk yield (MY) was recorded at each milking, and daily milk yield was calculated by summing the yields from consecutive morning and afternoon milkings. Milk samples were collected at both daily milkings and analyzed for fat, protein, and lactose using a CombiScope Fourier-transform infrared (FTIR) milk analyzer (Delta Instruments, Drachten, The Netherlands). Daily yields of milk fat, protein, and lactose were calculated from daily milk yield and the corresponding milk component concentrations.

Fat- and protein-corrected milk (FPCM) was calculated according to Opio et al. [13] as follows:

FPCM (kg/day) = milk yield (kg/day) × [0.337 + 0.116 × milk fat (%) + 0.060 × milk protein (%)]

### 2.5. Calculation of Methane Traits

Daily CH_4_ production was expressed as g/day. Methane yield was defined as CH_4_ production per unit of DMI (g/kg DMI), whereas methane intensity was used for CH_4_ expressed per unit of productive output, including milk yield, FPCM, and milk fat, protein, and lactose yields. The CH_4_/BW metric was reported separately as a body-weight-normalized metric (g/kg BW).

### 2.6. Statistical Analysis

Repeated measurements were averaged within each cow for each response variable before statistical analysis. The cow was considered the experimental unit. Breed differences between Holstein and Jersey cows were evaluated using independent two-sample *t*-tests in SAS Enterprise Guide 7.1 (SAS Institute Inc., Cary, NC, USA). Results are presented as arithmetic means and standard errors of the mean (SEM). Statistical significance was declared at *p* < 0.05.

As a sensitivity analysis, breed differences in BW, DMI, MY, FPCM, and CH_4_ traits were additionally evaluated using analysis of covariance with breed as a fixed effect and age and days in milk as covariates. Age and days in milk were averaged within cow across the retained measurement records before analysis. Parity was not included as an additional covariate because its distribution was strongly confounded with breed. The results of the sensitivity analysis are presented in Table S1.

### 2.7. Use of Generative Artificial Intelligence

Generative artificial intelligence (ChatGPT, OpenAI, GPT-5.6 Thinking; San Francisco, CA, USA) was used during manuscript preparation to assist with manuscript drafting, language refinement, and organization of the discussion. All AI-assisted content was reviewed and edited by the authors, who take full responsibility for the final content of the manuscript.

## 3. Results and Discussion

### 3.1. Breed-Specific Production and Milk Component Profiles

Holstein cows had greater BW and DMI than Jersey cows (both *p* < 0.0001; Table 2). Milk yield (*p* = 0.0003) and FPCM (*p* = 0.0055) were also greater in Holstein cows. The smaller difference in FPCM than in uncorrected MY reflects the contribution of milk fat and protein concentrations to composition-corrected milk output [5].

In contrast to their lower milk volume, Jersey cows had higher milk fat and protein concentrations than Holstein cows (*p* = 0.0002 and *p* = 0.0095, respectively). Lactose concentration did not differ significantly between breeds (*p* = 0.0534). The combination of higher per-cow milk production in Holstein cows and more concentrated fat and protein in Jersey milk can be interpreted as a contrast between production scale and milk nutrient density rather than as a uniform difference in productive performance [14,15]. A direct comparison conducted under common dietary and management conditions, a meta-analysis of Holstein–Jersey comparisons, and commercial herd data have consistently identified a separation between the greater milk output of Holstein cows and the higher milk component concentrations of Jersey cows [3,4,16].

The distinction between concentration and daily component output was also evident in the present results. Milk fat yield did not differ significantly between Holstein and Jersey cows (*p* = 0.2174). In contrast, protein yield (*p* = 0.0030) and lactose yield (*p* = 0.0003) were higher in Holstein cows. This pattern indicates that the higher fat concentration of Jersey milk largely compensated for its lower milk volume in terms of daily fat production under the conditions examined, whereas the higher protein concentration did not fully compensate for the lower milk volume in terms of daily protein yield. Commercial-herd studies have similarly shown that the more concentrated milk of Jersey cows does not necessarily result in higher component production per cow because absolute yield is determined jointly by milk volume and component concentration [17,18].

The extent to which component concentration offsets lower milk volume should not be regarded as a fixed breed relationship. Direct breed comparisons have shown that differences in milk yield, component production, and feed efficiency can vary with lactation stage and nutritional conditions [19,20,21]. In the present study, the lower milk volume of Jersey cows was largely offset for fat yield but not for protein or lactose yields. This component-dependent pattern indicates that milk composition and daily component production provide related but noninterchangeable descriptions of productive performance.

Overall, the results describe a volume–composition trade-off between the two breeds. Holstein cows produced more milk, FPCM, protein, and lactose per cow. Jersey cows produced milk with higher fat and protein concentrations, while their daily fat yield did not differ significantly from that of Holstein cows. This distinction provides the production context for the subsequent CH_4_ comparison because daily CH_4_ production and CH_4_ intensity expressed relative to corrected milk or milk components represent different metrics and should be interpreted separately [2,22].

### 3.2. Methane Production, Yield, and Intensity Traits

Daily CH_4_ production was higher in Holstein cows than in Jersey cows (*p* < 0.0001; Table 3). Consistent with the greater DMI of Holstein cows, methane yield (CH_4_/DMI) did not differ significantly between breeds (*p* = 0.7367).

Feed intake is a major determinant of absolute enteric CH_4_ production because greater DMI generally increases the amount of fermentable substrate entering the rumen [23]. Previous modeling and animal-level studies have identified DMI as a principal predictor of daily CH_4_ production and demonstrated a positive relationship between DMI and daily CH_4_ emissions [24,25]. The parallel differences in DMI and daily CH_4_ production observed in the present study are consistent with greater feed intake being associated with the higher daily CH_4_ production of Holstein cows. However, CH_4_/DMI was nearly identical between breeds in the present study, and no significant breed difference was detected.

Comparable breed patterns have been reported in both lactating and growing cattle. In a comparison of lactating cows, Holstein cows consumed 34% more DM and produced 22% more CH_4_ per day than Jersey cows, whereas CH_4_/DMI and CH_4_/FPCM were not significantly affected by breed [6]. Under Korean feeding conditions, Holstein heifers also produced more CH_4_ per day than Jersey heifers (136.99 vs. 105.53 g/day), while no significant breed difference was detected in CH_4_/DMI [26]. These results support the interpretation that a breed difference in per-cow CH_4_ production can occur without a corresponding difference in CH_4_ production per unit of feed intake.

Methane yield is also responsive to dietary composition and should not be regarded as fixed across feeding conditions. Dietary intervention studies have demonstrated that both intake- and milk-scaled CH_4_ traits respond to changes in forage and starch supply. In one study, increasing the forage proportion from 47 to 68% of dietary DM increased daily CH_4_ production and CH_4_ intensity per kilogram of energy-corrected milk (ECM), while DMI and ECM production remained unchanged [27]. In another respiration-chamber study, replacing fiber-rich grass silage with starch-rich corn silage reduced CH_4_ production by 11% per kilogram of DMI and by 8% per kilogram of FPCM without reducing milk production [28]. These findings show that dietary fiber and starch composition can alter intake- and corrected-milk-scaled CH_4_ traits even when feed intake or milk production changes little. Accordingly, the present CH_4_/DMI result is consistent with similar methane yield between Holstein and Jersey cows under the common dietary conditions evaluated here, while recognizing that CH_4_/DMI can vary with dietary composition.

Methane production per kilogram of BW was lower in Holstein cows than in Jersey cows (*p* < 0.0001). By comparison, no significant breed differences were detected in CH_4_ production per kilogram of milk yield (*p* = 0.6368) or FPCM (*p* = 0.2579). Recent prediction models and animal-level observational analyses have shown that BW, DMI, MY, and milk composition contribute differently to variation in daily CH_4_ production, yield, and intensity [29,30,31]. In the present dataset, the lower CH_4_/BW value in Holstein cows appears to reflect the much larger proportional breed difference in BW than in daily CH_4_ production rather than a clear advantage in production-related CH_4_ efficiency. Despite the higher daily CH_4_ production of Holstein cows, no corresponding increase was detected when CH_4_ was expressed per unit of milk or corrected milk.

A different pattern emerged when CH_4_ production was expressed relative to milk component yields. Methane intensity based on milk fat yield was lower in Jersey cows than in Holstein cows (*p* = 0.0323). This result was associated with lower daily CH_4_ production in Jersey cows and a milk fat yield that did not differ significantly between breeds. In contrast, no significant breed differences were detected in CH_4_ production per kilogram of milk protein yield (*p* = 0.2799) or lactose yield (*p* = 0.6253). Thus, the lower fat-yield-based CH_4_ value in Jersey cows was specific to the fat-yield denominator in the present dataset and was not reproduced across the other component-based traits. However, the nominal *p*-value for CH_4_/fat (*p* = 0.0323) did not meet the Bonferroni-adjusted significance threshold for the seven ratio-based CH_4_ traits evaluated in Table 3 (*α* = 0.0071).

Sensitivity analysis adjusting for age and days in milk generally supported the primary breed comparisons (Table S1). Breed effects remained significant for BW, DMI, MY, FPCM, daily CH_4_ production, and CH_4_/BW, whereas CH_4_/DMI, CH_4_/MY, CH_4_/FPCM, CH_4_/protein, and CH_4_/lactose remained nonsignificant. However, the breed difference in CH_4_/fat observed in the unadjusted analysis was inconclusive after adjustment for age and days in milk (*p* = 0.1117). The adjusted breed difference remained similar in magnitude to the unadjusted difference but was estimated with lower precision, with a 95% confidence interval that included zero (Table S1).

Component-based CH_4_ intensity is determined jointly by daily CH_4_ production and the yield of the milk component used as the denominator. Evidence from pasture-based commercial dairy farms illustrates how these two quantities can produce contrasting responses. As concentrate supplementation increased, daily enteric CH_4_ production increased, whereas CH_4_ production per kilogram of FPCM, milk fat yield, and milk protein yield decreased [32]. These findings support interpreting CH_4_/fat, CH_4_/protein, and CH_4_/lactose as output-specific measures rather than as interchangeable indicators of overall methane efficiency. Accordingly, the lower CH_4_/fat value of Jersey cows should be interpreted specifically in relation to milk fat output. The CH_4_/fat trait evaluated in the present study represents enteric CH_4_ normalized to cow-level milk fat yield and should not be interpreted as the carbon footprint of a processed dairy product.

### 3.3. Integrated Interpretation of Breed-Specific Methane and Production Profiles

Holstein cows had higher daily CH_4_ production, whereas no significant breed differences were detected for CH_4_/DMI or CH_4_/FPCM. The unadjusted analysis showed lower CH_4_ production per unit of milk fat yield in Jersey cows, although the corresponding breed difference was inconclusive after adjustment for age and days in milk (Table S1). Individual-cow distributions for selected CH_4_ traits are presented in Figure S1. These results do not indicate an overall CH_4_ advantage for either breed but show that the breed comparison differed according to the basis used to express CH_4_.

Several limitations should be considered when interpreting these findings. The study population consisted of 10 Holstein and 10 Jersey cows. Age, days in milk, and parity distributions also differed between the breed groups. Although sensitivity analysis adjusting for age and days in milk supported the principal breed differences in production traits and daily CH_4_ production, the CH_4_/fat difference was inconclusive after adjustment. Because parity was strongly confounded with breed, its independent effect could not be reliably separated from the breed effect in this dataset. The measurement periods were also not fully balanced between breeds across calendar time; therefore, period-related factors, such as seasonal conditions or TMR batch, could not be fully separated from breed effects in this dataset. Measurement period was not included as an additional covariate because the sensitivity analysis was conducted on cow-level averages across repeated measurement periods, so a single measurement-period value could not be assigned to each cow in this analytical framework. Several CH_4_ intensity traits did not differ significantly between breeds, although formal equivalence testing was not conducted. Because multiple methane traits were evaluated, the lower CH_4_/fat result should be regarded as exploratory and requires confirmation in larger direct breed comparisons.

## 4. Conclusions

Holstein cows had greater BW, DMI, milk yield, FPCM, and daily CH_4_ production than Jersey cows, whereas Jersey cows produced milk with higher fat and protein concentrations. Daily milk fat yield did not differ significantly between breeds, while protein and lactose yields were higher in Holstein cows. No significant breed differences were detected in CH_4_/DMI, CH_4_/MY, CH_4_/FPCM, CH_4_/protein, or CH_4_/lactose. In the unadjusted analysis, Jersey cows had lower CH_4_ production per unit of milk fat yield, but the breed difference was inconclusive after adjustment for age and days in milk. These findings indicate that the interpretation of breed differences in enteric CH_4_ depends on the basis used to express emissions. Accordingly, the component-based indices should be interpreted as exploratory animal-level measures rather than as alternatives to established CH_4_ production, yield, and corrected-milk intensity metrics. The results do not establish an overall methane advantage for either breed but identify distinct production and CH_4_ profiles that may be relevant to different dairy production objectives. Larger direct comparisons are needed to confirm the milk-fat-based result.

## Figures and Tables

**Table 1:** Ingredient and chemical composition of the total mixed ration.

Item	TMR
**Ingredients, % as fed**	
Commercial concentrate	31.72
Mixed hay (50% tall fescue and 50% orchardgrass)	11.53
Alfalfa hay	2.88
Timothy hay	2.88
Corn silage	43.25
Soybean meal	1.44
Whole cottonseed	2.88
Beet pulp	2.88
Vitamin–mineral mixture	0.19
Sodium bicarbonate	0.16
Yeast culture	0.16
Total	100.00
**Chemical composition, %**	
Dry matter	61.66 ± 4.87
Crude protein	13.63 ± 1.88
Ether extract	3.14 ± 0.41
Crude fiber	22.82 ± 2.08
Crude ash	7.72 ± 0.95
Neutral detergent fiber	49.98 ± 2.30
Acid detergent fiber	28.92 ± 2.38
Total digestible nutrients	58.86 ± 0.84

Total digestible nutrients (TDN) of the diet was estimated according to the National Research Council (NRC) [11].

**Table 2:** Production performance, milk composition, and milk component yields of Holstein and Jersey cows.

Trait	Unit	Holstein (*n* = 10)	Jersey (*n* = 10)	*p*-Value ^a^
**Production traits**				
BW	kg	688.50 ± 24.17	401.50 ± 12.90	<0.0001
DMI	kg/day	18.86 ± 0.83	13.68 ± 0.47	<0.0001
MY	kg/day	26.24 ± 1.53	18.33 ± 0.84	0.0003
FPCM	kg/day	27.93 ± 1.30	22.71 ± 1.02	0.0055
**Milk composition**				
Fat	%	4.69 ± 0.16	5.95 ± 0.22	0.0002
Protein	%	3.26 ± 0.06	3.64 ± 0.12	0.0095
Lactose	%	4.90 ± 0.06	4.78 ± 0.03	0.0534
**Milk component yields**				
Fat yield	kg/day	1.21 ± 0.06	1.10 ± 0.06	0.2174
Protein yield	kg/day	0.85 ± 0.04	0.67 ± 0.03	0.0030
Lactose yield	kg/day	1.28 ± 0.08	0.89 ± 0.05	0.0003

Values are presented as arithmetic means ± standard error of the mean (SEM). ^a^ *p*-values were derived from independent two-sample *t*-tests between Holstein and Jersey cows. Abbreviations: BW, body weight; DMI, dry matter intake; MY, milk yield; FPCM, fat- and protein-corrected milk.

**Table 3:** Methane production, yield, and intensity traits of Holstein and Jersey cows.

Trait	Unit	Holstein (*n* = 10)	Jersey (*n* = 10)	*p*-Value ^a^
**Absolute methane production**				
CH_4_ production	g/day	356.27 ± 7.61	258.78 ± 9.11	<0.0001
**Body weight and intake basis**				
CH_4_ per BW	g/kg BW	0.52 ± 0.01	0.65 ± 0.01	<0.0001
CH_4_ per DMI	g/kg DMI	19.30 ± 0.81	19.00 ± 0.35	0.7367
**Milk yield basis**				
CH_4_ per MY	g/kg milk	13.94 ± 0.72	14.54 ± 1.02	0.6368
CH_4_ per FPCM	g/kg FPCM	13.01 ± 0.60	11.80 ± 0.84	0.2579
**Milk component yield basis**				
CH_4_ per fat yield	g/kg fat	302.17 ± 14.56	246.66 ± 18.98	0.0323
CH_4_ per protein yield	g/kg protein	440.69 ± 26.08	400.00 ± 25.56	0.2799
CH_4_ per lactose yield	g/kg lactose	287.22 ± 17.27	301.24 ± 22.32	0.6253

Values are presented as arithmetic means ± standard error of the mean (SEM). ^a^ *p*-values were derived from independent two-sample *t*-tests between Holstein and Jersey cows and were not adjusted for multiple comparisons. Ratio-based CH_4_ traits were calculated as daily CH_4_ production divided by the corresponding BW, DMI, MY, FPCM, or milk component yield. Abbreviations: CH_4_, methane; BW, body weight; DMI, dry matter intake; MY, milk yield; FPCM, fat- and protein-corrected milk.

## Data Availability

The data presented in this study are available from the corresponding author upon reasonable request, subject to applicable institutional data-sharing procedures of the National Institute of Animal Science, Rural Development Administration.

## Supplementary Materials

The following supporting information can be downloaded at: https://www.mdpi.com/article/10.3390/ani16182898/s1, Figure S1: Methane production, yield, and intensity traits of Holstein and Jersey cows; Table S1: Sensitivity analysis of breed differences after adjustment for age and days in milk.

## Abbreviations

BW	Body weight
CH_4_	Methane
DM	Dry matter
DMI	Dry matter intake
ECM	Energy-corrected milk
FPCM	Fat- and protein-corrected milk
FTIR	Fourier-transform infrared
MY	Milk yield
NEL	Net energy for lactation
NRC	National Research Council
SEM	Standard error of the mean
TDN	Total digestible nutrients
TMR	Total mixed ration
