# Supplementary material for: Enteric Methane Emission and Milk Component Profiles of Holstein and Jersey Cows: A Respiration Chamber Study
Source: Animals (Basel). 2026 Sep 15;16(18):2898. doi: 10.3390/ani16182898 (PMC13603726; doi:10.3390/ani16182898)
Supplement: Supplementary file 1 [file animals-16-02898-s001.zip › animals-4514502-supplementary.pdf]

Table S1. Sensitivity analysis of breed differences after adjustment for age and days in milk.

Trait	Unit	Unadjusted p-value	Adjusted difference (Holstein − Jersey)	95% CI	Age- and DIM-adjusted p-value
BW	kg	<0.0001	186.21	148.36 − 224.07	<0.0001
DMI	kg/day	<0.0001	4.97	2.01 − 7.92	0.0026
MY	kg/day	0.0003	8.36	5.05 − 11.68	<0.0001
FPCM	kg/day	0.0055	4.64	1.25 − 8.03	0.0104
CH_4 production	g/day	<0.0001	75.44	42.85 − 108.03	0.0002
CH_4/BW	g/kg BW	<0.0001	−0.082	−0.132 − −0.032	0.0031
CH_4/DMI	g/kg DMI	0.7367	−0.64	−3.31 − 2.02	0.6155
CH_4/MY	g/kg milk	0.6368	−1.70	−4.05 − 0.64	0.1435
CH_4/FPCM	g/kg FPCM	0.2579	0.57	−1.73 − 2.88	0.6052
CH_4/fat yield	g/kg fat	0.0323	47.09	−12.22 − 106.40	0.1117
CH_4/protein yield	g/kg protein	0.2799	9.09	−82.49 − 100.67	0.8359
CH_4/lactose yield	g/kg lactose	0.6253	−40.41	−96.43 − 15.61	0.1457

Values represent the breed effect estimated by analysis of covariance with breed as a fixed effect and age and days in milk (DIM) as covariates. Adjusted differences are expressed as Holstein minus Jersey. The cow was considered the experimental unit (Holstein, n = 10; Jersey, n = 10). Unadjusted p-values are from the primary independent two-sample t-tests.

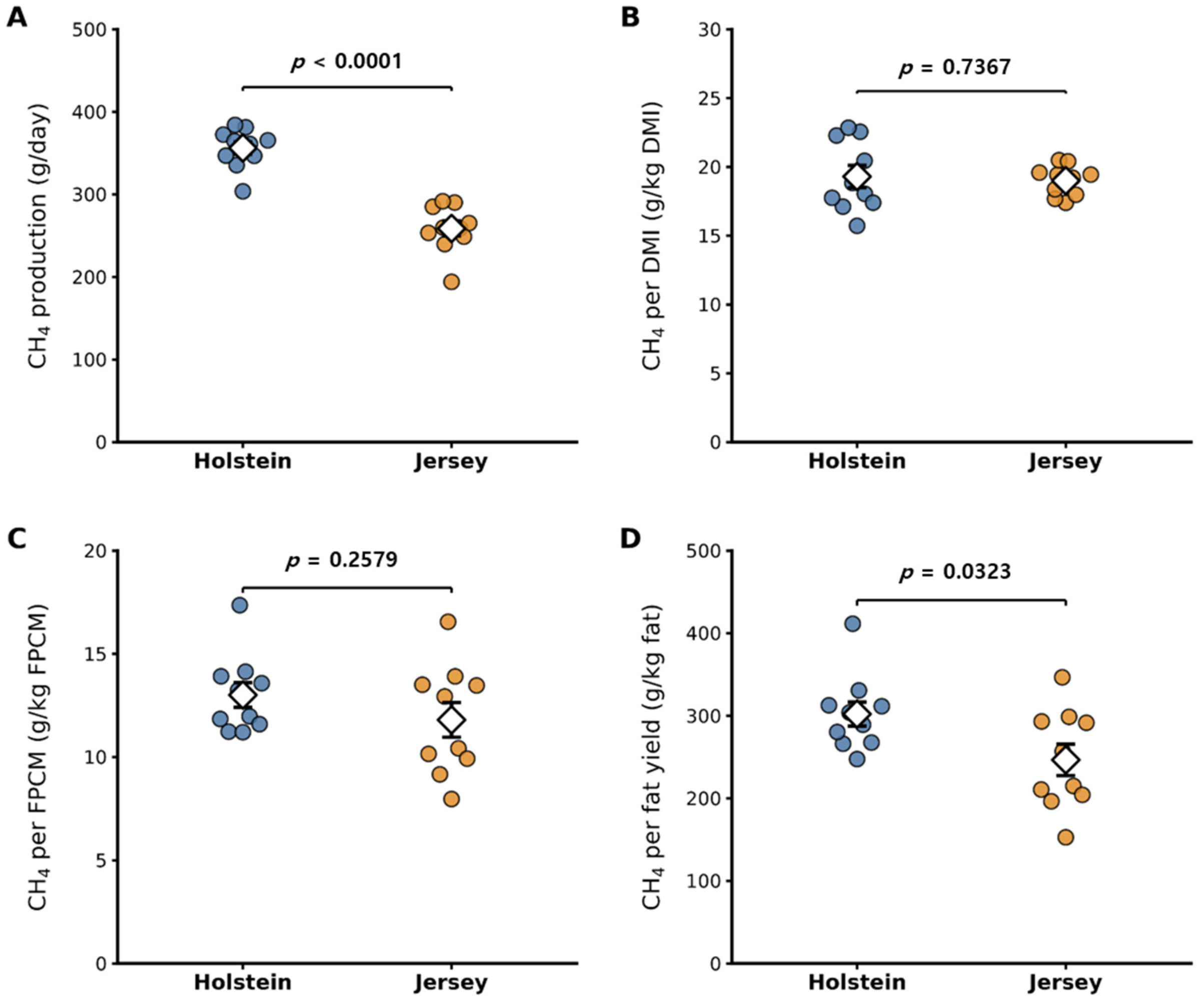

Supplementary Figure S1. Methane production, yield, and intensity traits of Holstein and Jersey cows. (A) Daily CH_4 production, (B) CH_4 production per unit of dry matter intake, (C) CH_4 production per unit of fat- and protein-corrected milk, and (D) CH_4 production per unit of milk fat yield. Colored circles represent the mean of repeated measurements for individual cows. White diamonds and error bars represent arithmetic means and standard errors of the mean (SEM), respectively. *p*-values were derived from independent two-sample t-tests between Holstein and Jersey cows. Holstein, n = 10; Jersey, n = 10.
